# Magnetic-field-assisted synthesis of anisotropic iron oxide particles: Effect of pH

**DOI:** 10.3762/bjnano.11.107

**Published:** 2020-08-17

**Authors:** Andrey V Shibaev, Petr V Shvets, Darya E Kessel, Roman A Kamyshinsky, Anton S Orekhov, Sergey S Abramchuk, Alexei R Khokhlov, Olga E Philippova

**Affiliations:** 1 Physics Department, Moscow State University, Leninskie Gory 1-2, 119991 Moscow, Russia; 2 REC “Functional Nanomaterials”, Immanuel Kant Baltic Federal University, A. Nevskogo ul. 14, 236041 Kaliningrad, Russia; 3 National Research Centre “Kurchatov Institute,” Akademika Kurchatova pl. 1, 123182 Moscow, Russia; 4 Moscow Institute of Physics & Technology, Institutskiy per. 9, 141700 Dolgoprudniy, Russia; 5 Institute of Advanced Energy Related Nanomaterials, Ulm University, Albert-Einstein-Allee 11, 89069 Ulm, Germany

**Keywords:** anisotropic nanoparticles, magnetic nanoparticles, magnetite, nanorods, transmission electron microscopy

## Abstract

The synthesis of magnetite (Fe_3_O_4_) nanorods using reverse co-precipitation of Fe^3+^ and Fe^2+^ ions in the presence of a static magnetic field is reported in this work. The phase composition and crystal structure of the synthesized material were investigated using electron diffraction, Raman spectroscopy, and transmission electron microscopy. It was shown that the morphology of the reaction product strongly depends on the amount of OH^−^ ions in the reaction mixture, varying from Fe_3_O_4_ nanorods to spherical Fe_3_O_4_ nanoparticles. Fe_3_O_4_ nanorods were examined using high-resolution transmission electron microscopy proving that they are single-crystalline and do not have any preferred crystallographic orientation along the axis of the rods. According to the data obtained a growth mechanism was proposed for the rods that consists of the dipole–dipole interaction between their building blocks (small hexagonal faceted magnetite nanocrystals), which are formed during the first step of the reaction. The study suggests a facile, green and controllable method for synthesizing anisotropic magnetic nanoparticles in the absence of stabilizers, which is important for further modification of their surfaces and/or incorporation of the nanoparticles into different media.

## Introduction

The research field dedicated to the synthesis and investigation of anisotropic magnetic nanomaterials has received much attention in the last years [1–3]. Among different magnetic nanomaterials, iron oxides and hydroxides are of particular interest because of their high magnetization capability, availability, low toxicity and environmental benigness [4–8]. These nanomaterials can be exploited in a variety of applications, including magnetic data storage [9], magnetic resonance imaging (MRI) [6,10–12], hyperthermia [6,13–15], magnetic separation [16], targeted drug delivery [6,16–19], lithium-ion batteries [8], preparation of hybrid organic–inorganic nanocomposites, and gels with polymer or surfactant-based matrices [20].

Among these applications, elongated particles (particularly nanorods) have many advantages over spherical nanoparticles [11,21–22]. Nanorods often have stronger magnetic properties and a larger length-scale of the locally induced magnetic field in comparison to nanospheres with a similar volume, providing an enhanced MRI contrast [11,19,23], higher specific adsorption rate in magnetic hyperthermia [13], and better separation efficiency in magnetic separation of immune cells [16]. Nanorods have been demonstrated to be effective in mechanically triggering tumoral cell death upon the application of low-frequency magnetic fields [24–25], which has not yet been observed in their spherical counterparts. In drug delivery, the elongated particles demonstrate a stronger binding to and an enhanced retention at the target sites [26] due to their larger contact area and multidentate interactions with the cell membranes. Thus, rod-like nanoparticles are preferred in many applications. However, their synthesis is more complicated than the synthesis of nanospheres since the cylindrical shape is less favorable due to its higher surface free energy.

So far, various methods for the preparation of iron oxide nanorods have been proposed [8,11,23,26–46]. These methods include co-precipitation [27–32], solvothermal [33] and hydrothermal [34] reactions, the polyol method [35], dehydration or reduction of precursor rod-like particles [8,11], sonochemical oxidation [36], thermal decomposition [23,26], sol–gel reactions [37], synthesis in worm-like surfactant micellar solutions [38–39] in the presence of shear [40], etc. Almost all of these methods involve either the use of a template or a stabilizing agent to induce a directional one-dimensional growth of rod-like nanoparticles. The most common agents used are surfactants [11,21,23,26,37], polymers [27,36] and other additives [8,35]. On the one hand, these approaches are advantageous since as-prepared nanoparticles covered by surfactant or polymer molecules become more stable and less susceptible to fast agglomeration. On the other hand, the nanoparticle surfaces become covered by these compounds, which are sometimes difficult to remove or replace.

Therefore, the elaboration of new and facile methods for synthesizing magnetic iron oxide nanorods, especially in the absence of additives, still poses a challenge. One of the proposed methods [28–30,37] is based on the exploitation of the magnetic properties of iron oxide. It consists of co-precipitating Fe^3+^ and Fe^2+^ ions upon exposure to an external magnetic field, which is used as a template for directional nanoparticle growth. Note that in the absence of a magnetic field, the same reagents yield spherical nanoparticles [30]. Hence, one can conclude that the magnetic field is responsible for the anisotropic growth. By using the magnetic-field-assisted synthesis, rod-like magnetite particles with different sizes and aspect ratios were prepared, including larger (micrometer-sized) [28] and smaller (nanometer-sized) [29–30,41] rods. Due to its simplicity and to the fact that neither toxic nor expensive reagents are used during the process, this method provides a facile and environmentally safe way to obtain anisotropic magnetic nanoparticles. However, there is still a lack of understanding of how the morphology and properties of synthesized nanoparticles can be changed in a controllable manner. In addition, no study has been performed so far to elucidate how the synthesis conditions influence the nanoparticle shape, size, and crystal structure.

Recent studies [14,31–32,42] showed that one of the key parameters that controls the iron oxide nanoparticle properties is the pH during synthesis. This is due to the fact that the OH^−^ ion concentration in the reaction mixture controls the reaction rate and, therefore, the nanocrystal growth mechanism. For instance, Ahn et al. [31] studied the formation of magnetite nanoparticles by performing co-precipitation assays using different molar ratios (*R*) of ammonia (OH^−^ ion source) and iron ions. The authors showed that Fe_3_O_4_ particles were not produced by a direct reaction of Fe^3+^, Fe^2+^, and OH^−^ ions, but rather via formation of several iron oxyhydroxides (including goetite or lepidocrocite) and their further transformation into magnetite. The reaction pathway was strongly dependent on *R* and also on the rate in which the base was added. At the same time, the effect of pH on the templated directional synthesis of iron oxide as well as the formation of nanorods within this context are yet to be determined.

In this work, for the first time, the effect of pH on the magnetic-field-assisted synthesis of iron oxide nanoparticles was investigated. The results show that different nanostructures were formed upon varying the initial pH of the reaction mixture: spheres were obtained at a highly alkaline pH whereas rods were obtained at a slightly acidic pH. Thus, we determined the optimum pH for nanorod preparation. High-resolution transmission electron microscopy (HRTEM) results showed that the synthesized nanorods were single crystals formed by the magnetic-field-assisted growth of small nanocrystals, whereas some amount of these nanocrystals still persists in the reaction products.

## Results and Discussion

### Main synthetic routes

**Figure 1:**
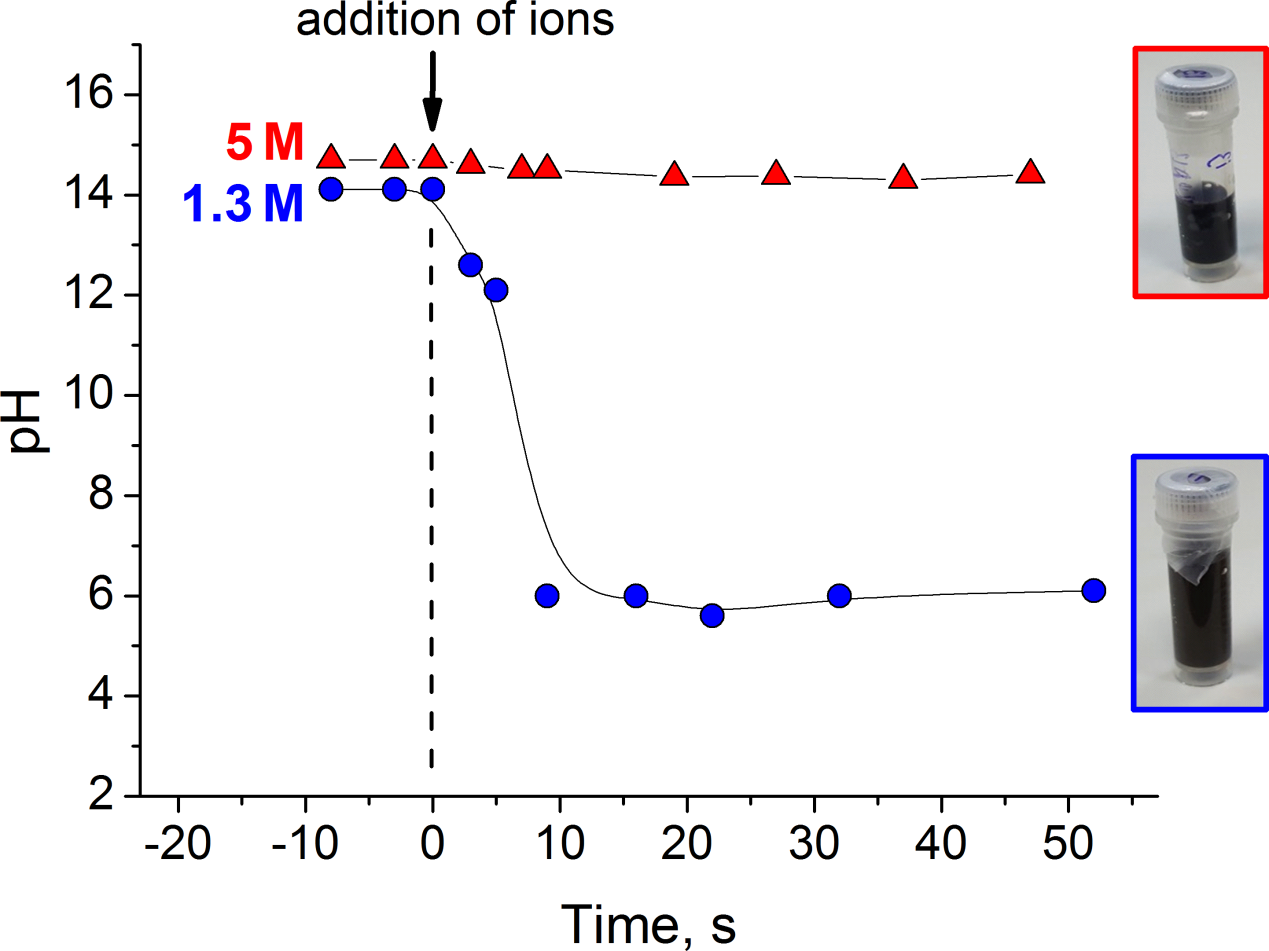
Temporal variation of pH when 2 mL of the ion mixture (1 M FeCl_3_ + 0.5 M FeSO_4_ in 0.1 M HCl) is added to 5 mL of NaOH solutions of two different concentrations: 5 M (triangles) and 1.3 M (circles), under exposure to a magnetic field of 0.4 T and at 20 °C.

The effect of pH on the magnetic-field-assisted synthesis of iron oxide nanoparticles was studied by adding 2 mL of the iron ion solution (1 M FeCl_3_ and 0.5 M FeSO_4_ in 0.1 M HCl) to 5 mL of NaOH solution with different concentrations (5 and 1.3 M). The different NaOH concentrations correspond to different molar ratios of OH^−^ ions with respect to the total amount of iron ions (Fe^3+^ and Fe^2+^) in the mixture. The synthesis conditions were characterized according to the molar ratio of ions in the mixture as:


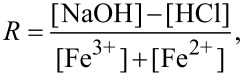


where [NaOH], [HCl], [Fe^3+^] and [Fe^2+^] are the equilibrium concentrations of the corresponding species. In the case of the 5 M NaOH feeding solution, there is a high excess of hydroxyl ions (*R* = 8), which is confirmed by the small decrease in pH during synthesis (Figure 1) as a result of OH^−^ consumption by the iron ions in the reaction. As a consequence, at the end of the reaction, the pH value is still highly basic (pH ≈ 14.4). For the 1.3 M NaOH feeding solution, the amounts of hydroxyl and iron ions in the mixture are comparable (*R* = 2.1). In this case, the decrease in pH is more pronounced and, when the reaction is completed, it becomes slightly acidic (pH ≈ 6.1), meaning that all the hydroxyl ions were consumed during synthesis (Figure 1). Figure 1 shows that when the iron ion solution (pH 1) is added, the pH value decreases rapidly (within several seconds) and then stays nearly constant until the end of the synthesis (2–2.5 h). During the first several seconds, the color of the reaction mixture changes to black (Figure 1). This is consistent with the fact that the crystal nucleation happens almost instantly [6,43]. After that, only changes or reorganization of the crystalline structure of the obtained nanostructures take place [14].

Two synthesis routes (at *R* = 2.1 and *R* = 8) will be considered in more detail. In addition, the effect of the ratio *R* between OH^−^ and iron ions on the morphology of the synthesized nanostructures will be elucidated. First, the synthesis using comparable amounts of reacting ions (*R* = 2.1) will be described and then compared with the case in which hydroxyl ions are in large excess (*R* = 8).

### Comparable amounts of hydroxyl and iron ions (*R* = 2.1)

**Figure 2:**
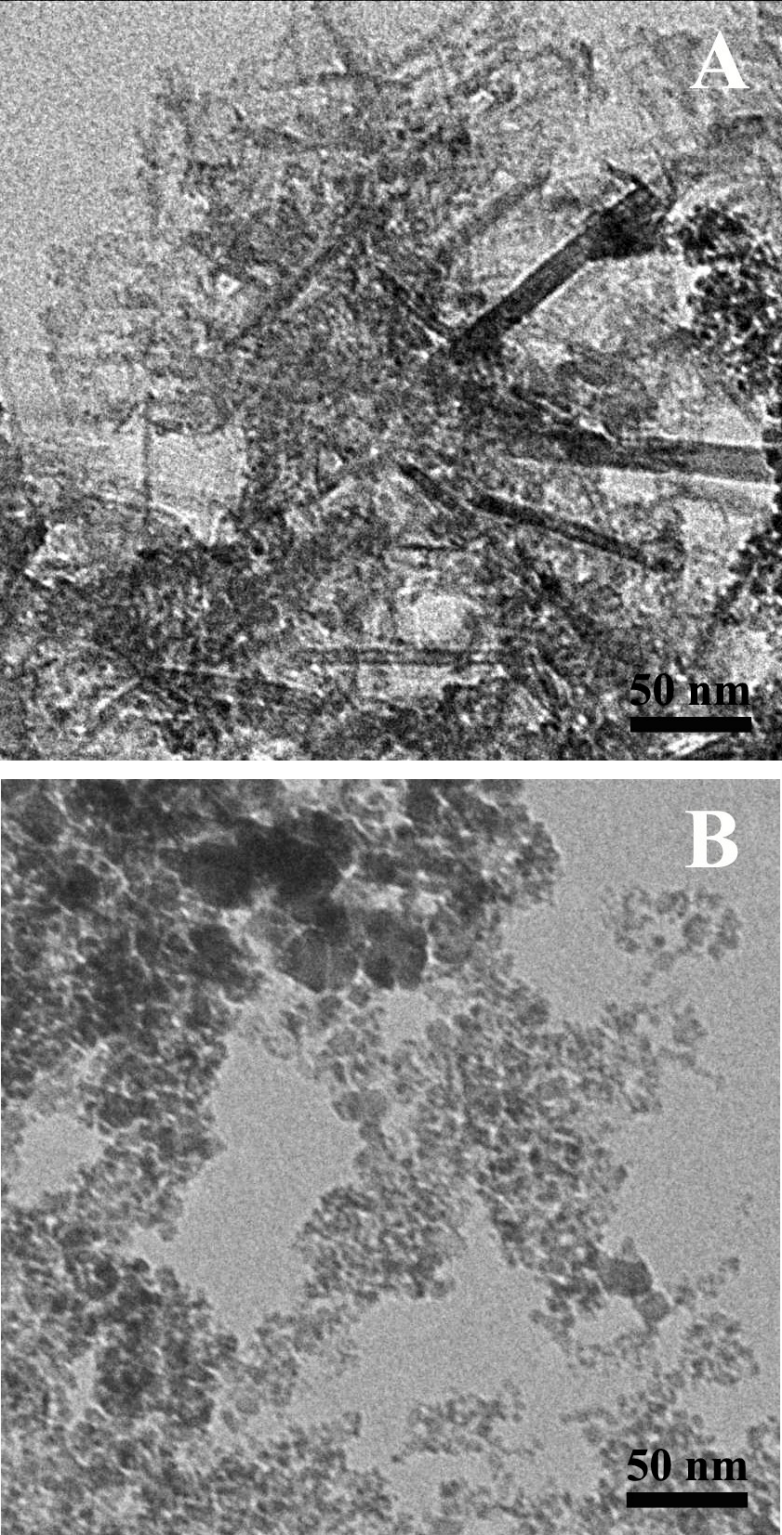
TEM micrographs showing nanostructures synthesized under a magnetic field of 0.4 T and at 20 °C at different molar ratios *R* of OH^−^ ions with respect to the total amount of iron ions (Fe^3+^ and Fe^2+^) in the mixture: *R* = 2.1 (A) and *R* = 8 (B).

**Figure 3:**
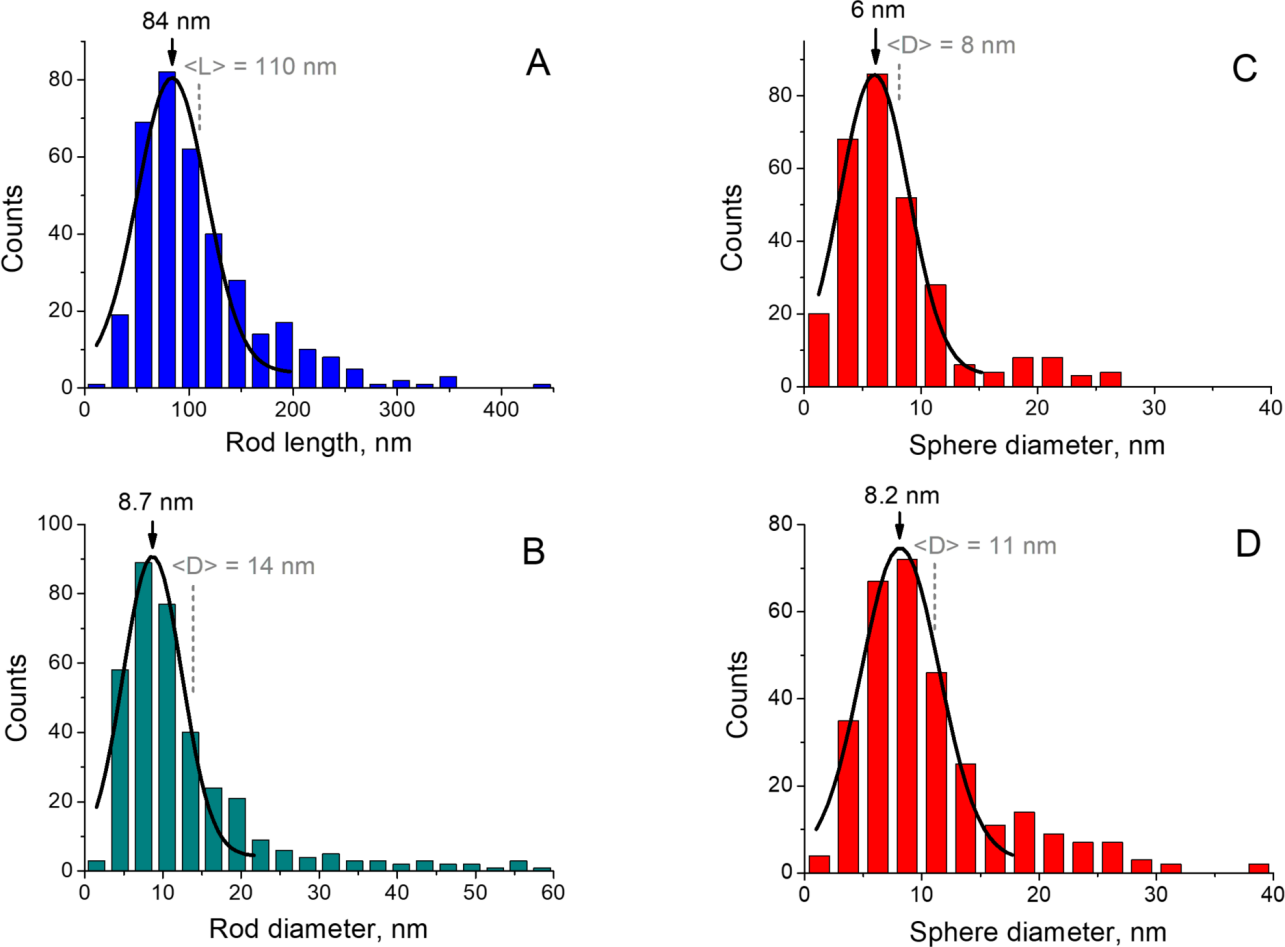
Size distribution histograms of nanoparticles obtained from TEM micrographs at different molar ratios *R*. (A) Length of the rods synthesized at *R* = 2.1. (B) Diameter of the rods synthesized at *R* = 2.1. (C) Diameter of the spheres coexisting with the rods at *R* = 2.1. (D) Diameter of the spheres synthesized at *R* = 8.

According to the transmission electron microscopy (TEM) images (Figure 2A), the black precipitate formed under these conditions consists mainly of rod-like nanoparticles and some spherical particles. The rods have a mean length of 110 nm (Figure 3A), a mean diameter of 14 nm (Figure 3B) and an aspect ratio of 8. The spheres, on the other hand, have a smaller average diameter of 8 nm (Figure 3C). In the absence of magnetic field, only spheres are formed under these conditions [30].

**Figure 4:**
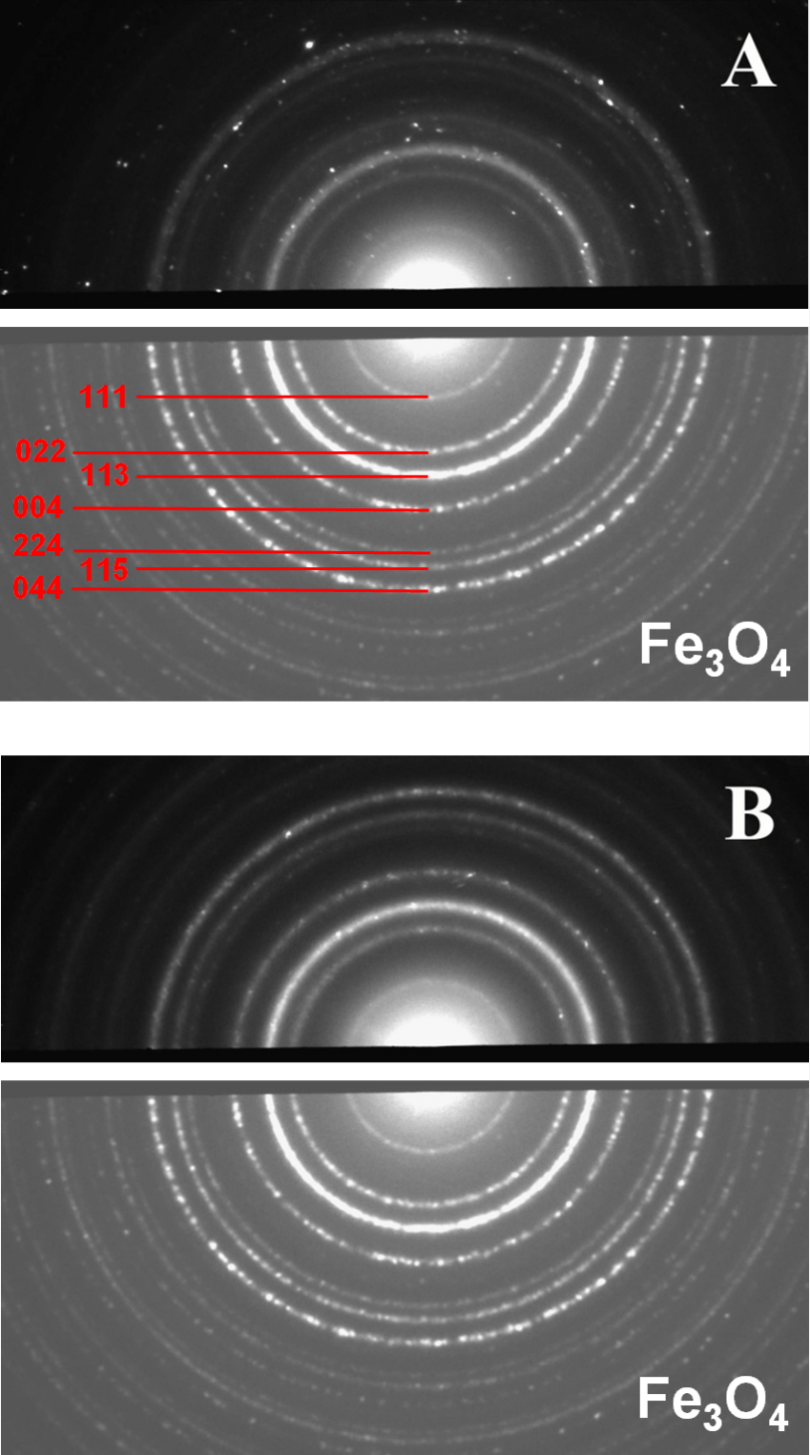
2D electron diffraction patterns of nanoparticles synthesized under a magnetic field of 0.4 T at 20 °C and at different molar ratios *R*: *R* = 2.1 (A) and *R* = 8 (B). The bottom part of the diffractogramms represent the diffraction pattern of the reference sample (commercial Fe_3_O_4_). Attribution of the diffraction rings for the reference sample was obtained from the International Centre for Diffraction Data (ICDD) database.

Figure 4A shows the electron diffraction data obtained from nanoparticles prepared at *R* = 2.1. For comparison, the diffraction pattern of the commercial magnetite Fe_3_O_4_ (reference sample) is displayed in the lower pannel of Figure 4A. It is seen that all the diffraction rings present in the experimental sample coincide with the rings in the reference pattern, suggesting the formation of a magnetite crystalline structure. Note that both rings and point reflexes are seen in the diffraction pattern (Figure 4A). This is due to the coexistence between small spherical particles (giving diffraction rings) and larger rod-like particles (contributing mostly to point reflexes), as confirmed by TEM images taken in the dark-field mode. Almost all of the point reflexes are localized in the magnetite rings, confirming that spherical and rod-like nanoparticles have the same crystal structure. Some of these point reflexes correspond to very weak rings that are scarcely seen even in the reference diffraction pattern.

**Figure 5:**
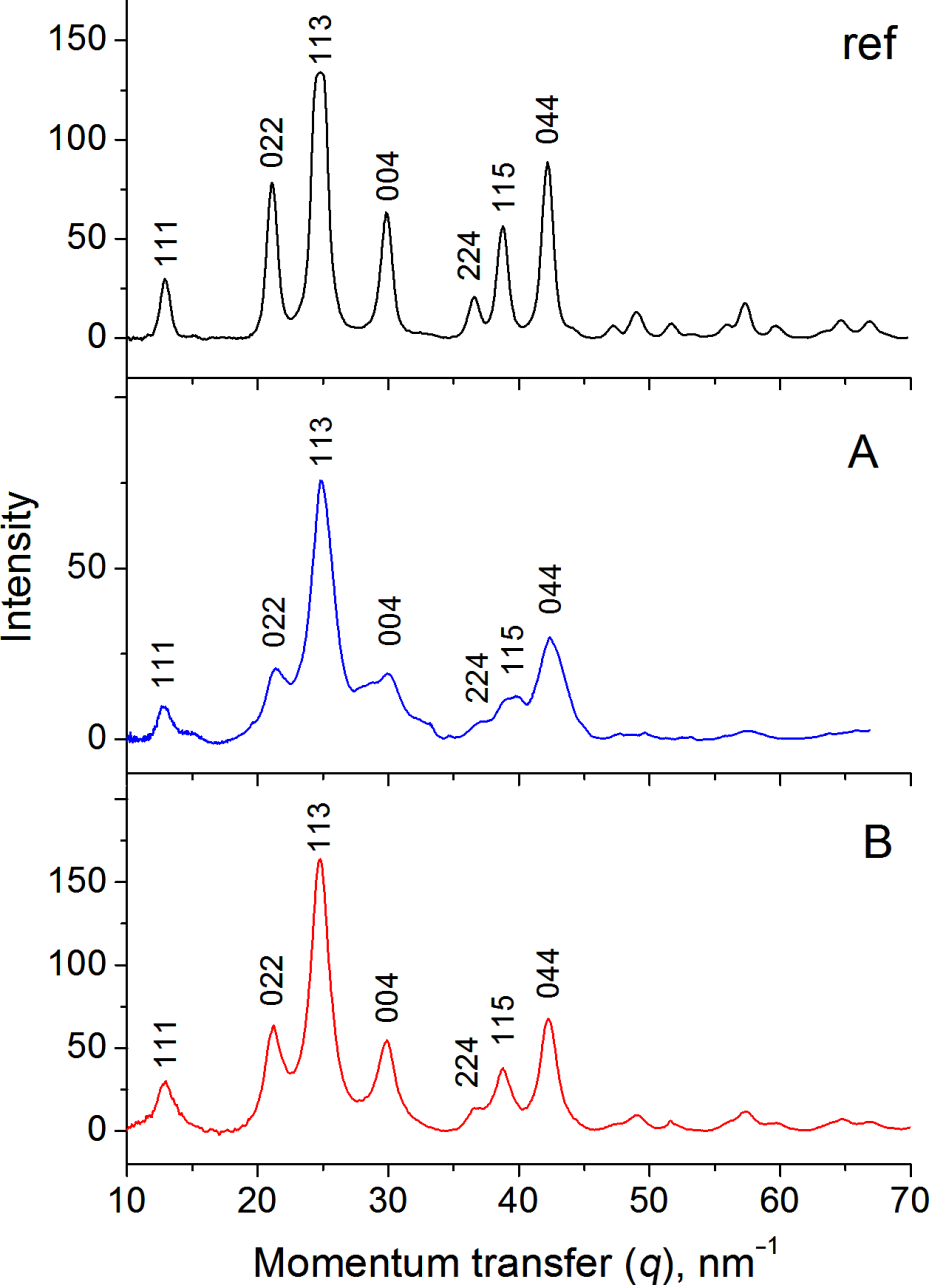
1D electron diffraction patterns obtained by the radial averaging of 2D patterns (Figure 4) for the reference Fe_3_O_4_ sample (ref) and for the samples synthesized at different molar ratios *R*. (A) *R* = 2.1 and (B) *R* = 8, under a magnetic field of 0.4 T and at 20 °C. The numbers near the peaks indicate the corresponding reflexes.

In order to get detailed information about the crystal structure of the synthesized nanoparticles, a 1D electron diffraction pattern was obtained by radial averaging the 2D patterns and the results are presented in Figure 5A. For comparison, the corresponding pattern for the reference sample is shown in the top panel of Figure 5. The parameters of the diffraction peaks are summarized in Table 1. Figure 5 shows that the areas of all peaks from the experimental samples coincide with the reference sample, indicating an absence of texturing in the rod-like nanoparticles. Consequently, since most of the rods are oriented perpendicular to the electron beam, it can be assumed that there is no preferential crystal plane oriented perpendicular to the rod length. At the same time, the full width at half maximum (FWHM) of all peaks of the experimental samples is higher than in the reference sample, which is in an accordance with the Scherrer equation [44], and indicates a smaller nanoparticle size (8 nm) than in the reference sample (20–50 nm).

**Table 1:** Parameters of the diffraction peaks obtained from the radial averaging of 1D electron diffraction patterns (Figure 5) [45].

Reflex	*d*-spacing, Å	Relative peak area				Width, nm^−1^		
		
		Reference	Rods + spheres (*R* = 2.1)	Spheres (*R* = 8)		Reference	Rods + spheres (*R* = 2.1)	Spheres (*R* = 8)

111	4.847	10	7	13		0.67	1.03	1.40
022	2.968	37	36	38		0.86	2.27	1.77
113	2.531	100	100	100		1.24	1.73	1.72
004	2.099	31	48	36		0.93	4.04	2.16
224	1.714	8	7	5		0.84	1.34	0.93
115	1.616	27	17	20		0.89	1.80	1.66
044	1.484	45	49	34		0.92	2.12	1.43

**Figure 6:**
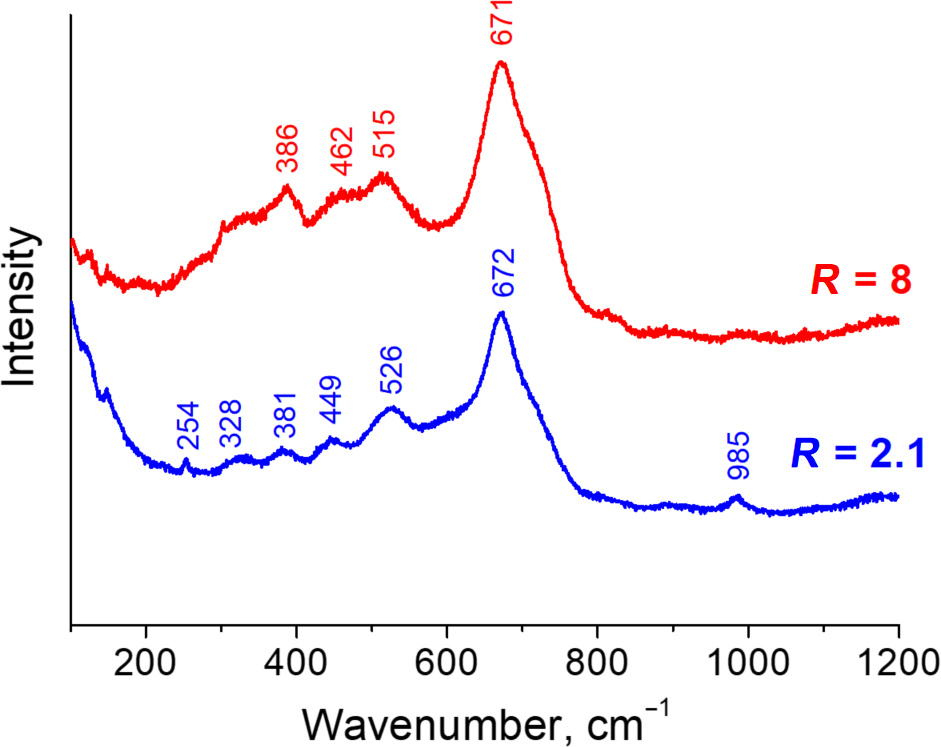
Raman spectra of the samples synthesized at different molar ratios *R*: *R* = 2.1 (blue curve) and *R* = 8 (red curve), under a magnetic field of 0.4 T and at 20 °C.

It is well known that distinguishing between magnetite (Fe_3_O_4_) and maghemite (γ-Fe_2_O_3_) only using the diffraction technique is not straightforward. However, these samples can be easily distinguished by Raman scattering, since Fe_3_O_4_, γ-Fe_2_O_3_ and other iron oxides and hydroxides have very different vibrational bands [46–47]. The Raman spectrum of the sample synthesized at *R* = 2.1 is shown in Figure 6 (blue curve). The data shows that the main band (672 cm^−1^) coincides with the main vibrational mode of magnetite (A_1g_) [48–49], thus proving that Fe_3_O_4_ nanoparticles were synthesized through this route. It should be noted that, due to the wide peaks in the Raman spectrum and to the presence of additional smaller peaks, there might be other iron oxides in the sample [50], although magnetite is prevalent. This is confirmed by energy-dispersive X-ray (EDX) spectrum taken from the region of the sample where rod-like objects are present. The results demonstrate that the ratio of Fe to O atoms is equal to ≈0.78, supporting the formation of magnetite during the synthesis process (Supporting Information File 1, Figure S1 and Table S1).

**Figure 7:**
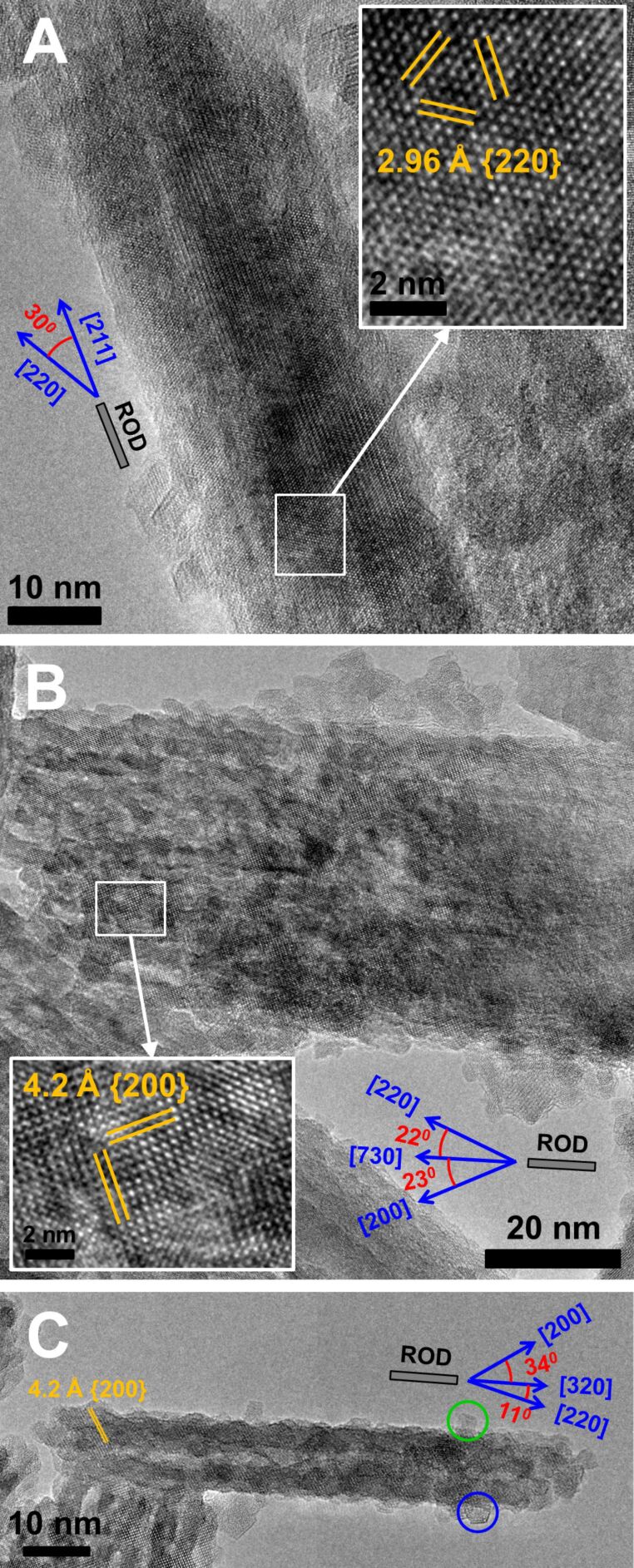
HRTEM micrographs of Fe_3_O_4_ nanorods synthesized at *R* = 2.1 under a magnetic field of 0.4 T and at 20 °C. (A) The {111} plane is given and the {220} plane spacing (2.96 Å) is identified in the magnified part of the rod (inset). (B) The {001} plane is given and the {200} plane spacing (4.2 Å) is identified in the magnified part of the rod (inset). (C) The {001} plane is given and the {200} plane spacing (4.2 Å) is identified in the nanorod. Interplanar distances of 2.96 and 4.2 Å are assigned to the {220} and {200} planes of the Fe_3_O_4_ crystal, respectively. Hexagonal (blue circle) and cubic (green circle) primary particles are attached to the surface of the rod.

In order to get deeper insight into the crystalline structure of the nanoparticles and their growth mechanism under magnetic field exposure, HRTEM images were obtained. Figure 7 shows HRTEM images of different nanorods. It is seen that the synthesized nanorods are single crystals. From the interatomic distances, a few magnetite crystal planes can be identified. For instance, Figure 7A shows distinct lattice fringes with a spacing of 2.96 Å, which corresponds to the Fe_3_O_4_ {220} crystal plane. The major axis of this nanorod is in the [211] direction. The image lies in the {111} plane. From the [111] direction, the magnetite crystal lattice is seen as hexagonal. Figure 7B and Figure 7C demonstrate a spacing of 4.2 Å, corresponding to the Fe_3_O_4_ {200} crystal planes. These images lie in the {001} plane and a cubic lattice is seen from the [001] direction.

In addition, Figure 7 shows that there is no preferential crystal axis positioned directly along the rod length, which is in agreement with the diffraction data presented previously. Indeed, from the angle between the [220] axis and the rod axis different axes were identified, including those with high Miller indices pointing in the direction of the rod axis: [211] (Figure 7A), [730] (Figure 7B), and [320] (Figure 7C).

**Figure 8:**
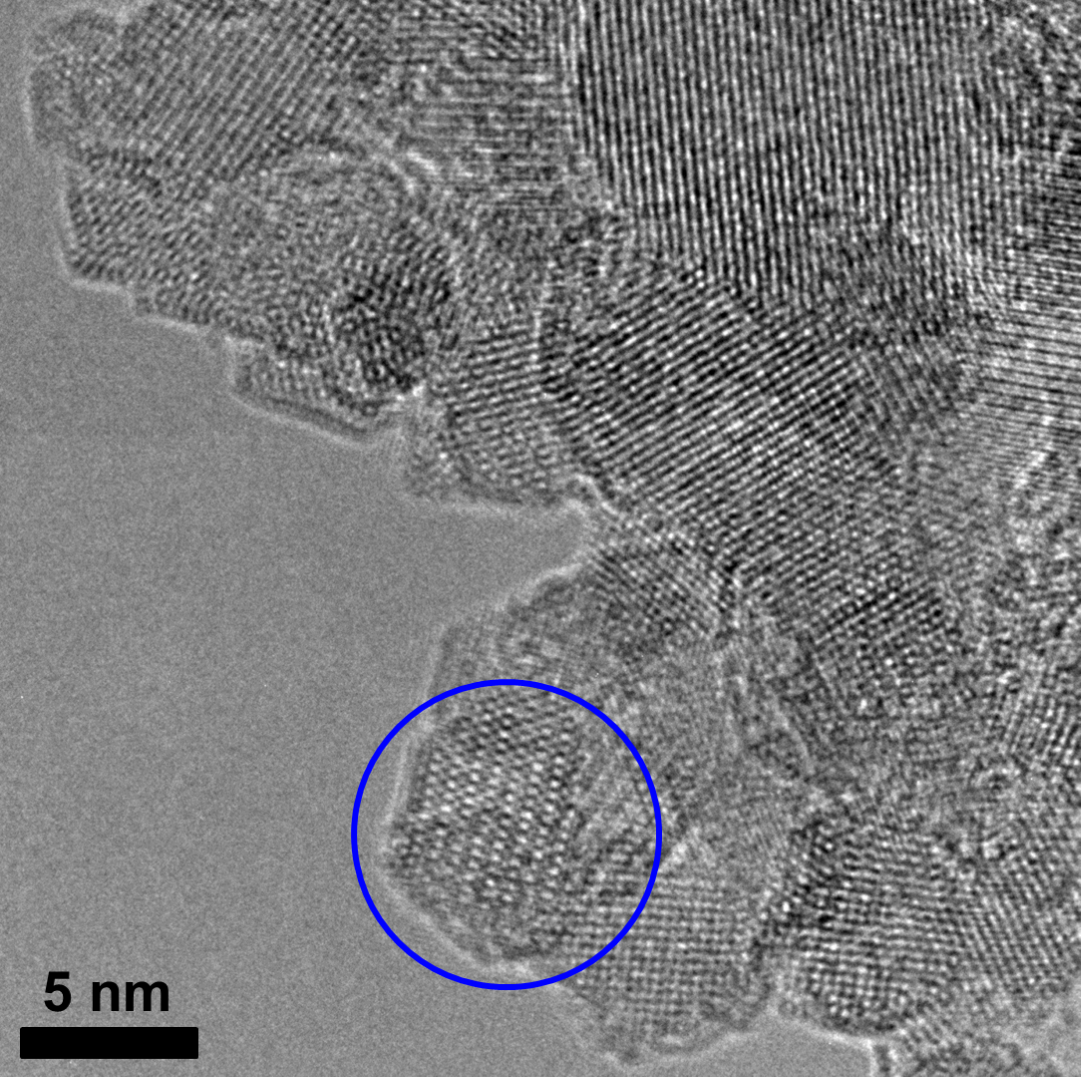
HRTEM micrograph of hexagonal iron oxide nanoparticles synthesized at *R* = 2.1 under a magnetic field of 0.4 T and at 20 °C.

Figure 8 shows the HRTEM image of small nanoparticles coexisting with the rods. Many of them have a hexagonal shape and are single crystalline. It is important to note that most of the rods are covered by the same nanoparticles (highlighted by a blue circle in Figure 7c). Similar pictures depicting many primary nanoparticles attached to the surface of a larger magnetite particle were recently observed in spherical nanoparticles [9]. This was considered as evidence for magnetite nucleation and the growth mechanism, consisting of the aggregation of primary nanoparticles.

As to our system, according to HRTEM data, one can propose the following growth mechanism of magnetic nanorods. Since there is no one preferential crystal plane oriented perpendicular to the rod length, the rod does not grow due to the attachment of individual atoms. Most likely, the growth process starts when small spherical or hexagonal nanocrystals (i.e., primary nanocrystals) [51] are formed, which collectively orient and move according to the external field applied [52], forming chain-like or column-like structures [53–54]. The mean diameter of the primary nanocrystals is 8 nm (Figure 3C and Figure 7C), which is only slightly larger than the minimum diameter of the magnetite particles (6.5 nm) [28], which is necessary for the magnetic force to overcome the Brownian motion of the particles. Therefore, the 8 nm primary nanocrysals are able to form chain-like structures upon the action of an external magnetic field. Furthermore, the magnetic field can trigger the aggregation of chain-like structures into column-like structures (Figure 7C), thus contributing to the growth of the aggregates. Note that at comparable amounts of hydroxyl and iron ions (*R* = 2.1) the magnetite particles are only slightly charged, since the resulting pH of the solution (pH 6.1) is close to the isoelectric point of magnetite particles (6.0–6.7 [6,22,28]). As expected, the low charge of the nanoparticles favors their aggregation and contact crystallization.

Hence, under a magnetic field the primary nanocrystals bind to each other and position themselves in a column-like orientation, which gives a rod-like shape to the aggregate. Further recrystallization of the aggregates leads to the formation of single-crystal nanorods (Figure 7). The formation of single-crystal nanorods may be facilitated by the hexagonal shape of the primary nanocrystals, so that the adjacent nanocrystals can mutually orient along their facets that is along the same crystal plane {220}. The attachment of the single-crystal nanorods releases the interfacial tension [22], thus reducing the free energy. However, if the bound nanocrystals are oriented along different crystal planes within the aggregates, the crystalline structure should be reorganized in order to give a single-crystal rod. The presence of primary nanocrystals building the rod-like particles (shown in Figure 7c) favors the proposed model of particle growth by aggregation and recrystallization of primary nanocrystals. Nonetheless, one cannot fully neglect direct crystal growth by the accretion of atoms, which can help the construction of single rod-like crystals on the basis of column-like aggregates of particles acting as a template. The growth of rods by aggregation and recrystallization of primary particles was previously described for micrometer-sized rod-like magnetite particles synthesized by co-precipitation under different conditions [28]. In that case [28], the rods were composed of primary particles bound together. In our system, some primary particles are attached to the surface of the rods; however, the main body of the rods is single-crystalline (Figure 7).

Thus, at comparable amounts of OH^−^ and iron ions (Fe^3+^ and Fe^2+^) (*R* = 2.1) in the mixture, the presence of a magnetic field has a direct influence on the morphology of the nanoparticles, leading to the formation of rod-like single-crystalline particles. The same mixture of rods and spheres was observed at slightly higher value of *R* = 2.4 obtained with a 1.45 M NaOH feeding solution (Supporting Information File 1, Figure S2A).

### Excess of hydroxyl ions (*R* = 8)

**Figure 9:**
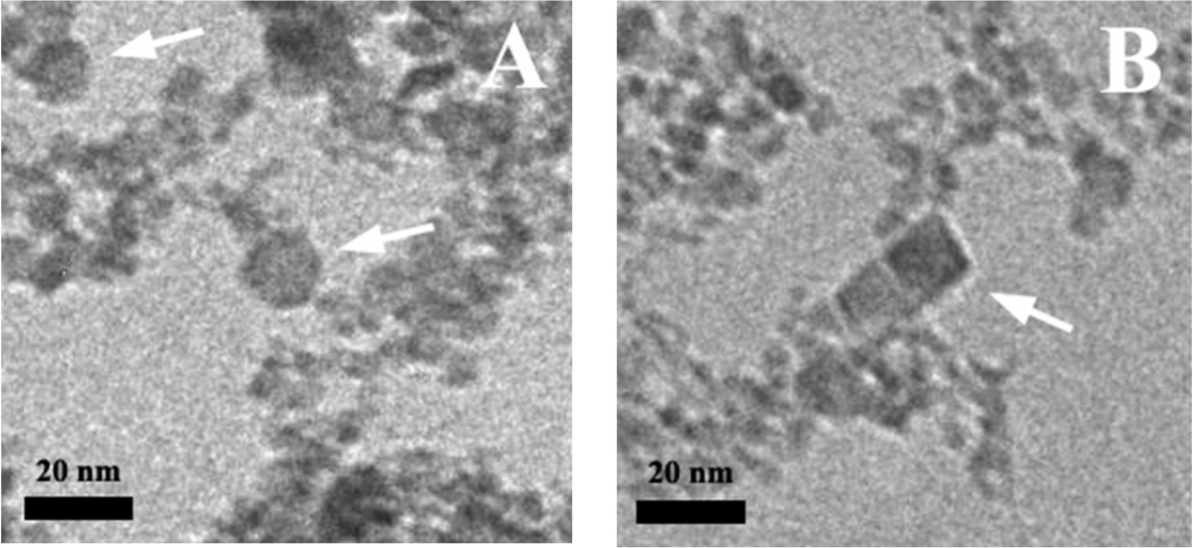
TEM micrographs of iron oxide nanoparticles synthesized at *R* = 8 under a magnetic field of 0.4 T and at 20 °C. Hexagonal (A) and cubic (B) nanoparticles are identified with arrows.

Under this synthesis route, the precipitate formed in the reaction mixture is also black and magnetic. TEM data (Figure 2B) show that, in this case, only isotropic nanoparticles are synthesized and their mean diameter is 11 nm (Figure 3A). The main nanoparticle fraction has a diameter of 8.2 nm, similar to the spherical particles coexisting with the rods prepared under the first synthesis route at *R* = 2.1 (8 nm, Figure 3A). Only a small fraction of the larger polygonal nanoparticles (≈17.5 nm) is seen in TEM images and the corresponding histogram. TEM micrographs show particles with different morphologies: spherical, hexagonal and cubic (Figure 9). The spherical nanoparticles result from isotropic crystal growth whereas hexagons and cubes are formed upon anisotropic growth, preserving the {111} or {100} crystal facets, respectively [23]. Note that the {100} facet is the most stable one in the spinel structure, having the lowest energy, whereas the {111} facet has the highest density of iron ions.

2D electron diffraction data (Figure 4B) and Raman spectrum (Figure 6, red curve) confirm the formation of the magnetite phase. Contrary to the case in which rod-like and spherical nanoparticles are mixed (Figure 4A), only rings are present in the 2D electron diffractograms. The presence of rings but not point reflexes is consistent with the size of nanoparticles being smaller than the diameter of the illuminated area (2 μm) and with their random orientation. Data obtained from the radial-averaged diffraction pattern (Figure 5, Table 1) show that the areas of all peaks generally coincide, within the experimental error, with the values for the reference sample, indicating that there is no preferential orientation of the nanoparticle crystal planes on the microscopy grid. At the same time, the width of the peaks decreases (Table 1) in comparison with the first synthetic route (*R* = 2.1), which is in agreement with the fact that the spherical nanoparticles synthesized at *R* = 8 are larger than the spherical particles coexisting with the rods at *R* = 2.1.

It should be noted that Fe_3_O_4_ spherical nanoparticles with similar size are obtained under the same conditions but in the absence of a magnetic field (corresponding TEM micrograph and size distribution are presented in Supporting Information File 1, Figure S3). Therefore, under this synthesis route, the magnetic field has almost no effect on the morphology of the synthesized nanostructures. This is probably due to the highly charged surface of the particles, since at the excess of hydroxyl ions the resulting solution has very high pH (pH ≈ 14), far exceeding the isoelectric point of magnetite particles (6.0–6.7 [6,21,27]). This is confirmed by the fact that similar spherical nanoparticles and no rod-like structures are seen at lower *R* values (2.8 and 3.3), obtained with 1.7 M and 2 M NaOH feeding solutions (Figures S2B and S2C, Supporting Information File 1), but giving rather high pH values of the resulting solution (9 and 12, respectively) at which magnetite is strongly charged. Therefore, when primary nanoparticles are highly charged their mutual aggregation under a magnetic field is prevented and their preferential growth seems to happen via a direct crystal growth mechanism [55].

## Conclusion

In this paper, we have shown that pH, which controls the molar ratio of OH^−^ to iron ions, has a direct influence on the magnetic-field-assisted synthesis of iron oxide nanostructures. At a high OH^−^ content, the particles are spherical, independent of the presence of a magnetic field, which is due to the high charge of their surface, preventing their magnetic-field-induced aggregation. At OH^−^ concentrations comparable to the concentration of iron ions, when the nanoparticles are only slightly charged, the nanoparticles are able to align along the magnetic field, leading to the formation of magnetite single-crystal nanorods. The crystal structure of the main products in all cases was identified using electron diffraction and confirmed by Raman spectroscopy. Diffraction data combined with HRTEM micrographs showed that magnetite nanorods are single crystalline and have no preferred crystallographic orientation along the rod axis. We propose that the formation of such cylindrical structures is due to the dipole–dipole interaction between their building blocks (small hexagonal faceted magnetite nanocrystals), which are formed during the first step of the reaction. The findings of this paper open a route for the precise control of magnetite nanorod morphology using a simple magnetic-field-assisted synthesis method without any stabilizers.

## Experimental

### Materials

Iron(III) chloride hexahydrate (FeCl_3_·6H_2_O, purity >97%) and iron(II) sulfate heptahydrate (FeSO_4_·7H_2_O, purity >99%) were purchased from Sigma-Aldrich (St. Louis, MO, USA). Sodium hydroxide (purity >98%, residual water content <15%) was obtained from Acros (Fair Lawn, NJ, USA), and hydrochloric acid was purchased from ZAO Uralchiminvest (Ufa, Russia) as a titration standard for the preparation of a 0.1 M solution. All chemicals were used without further purification. The solutions were prepared in distilled deionized water purified by the Millipore Milli-Q system (Burlington, MA, USA).

### Synthesis of nanoparticles

A scheme of the experimental setup used for the nanoparticle synthesis is presented in Figure S4 (see Supporting Information File 1). The iron ion solution with a Fe^3+^:Fe^2+^ molar ratio of 2:1 was prepared by dissolving 1 M FeCl_3_ and 0.5 M FeSO_4_ in 0.1 M HCl under magnetic stirring. In order to prevent the oxidation of Fe^2+^ ions, the solution was degassed by purging with argon for 5 min. For the synthesis, the reverse co-precipitation method was used in a similar manner as previously described [30]: 2 mL of the iron ion solution were quickly added to a 5 mL of the NaOH solution of an appropriate concentration under stirring, upon a static magnetic field of 0.4 T and constant purging of argon.

The magnetic field was applied during the whole synthesis procedure. The intensity of the magnetic field (0.4 T) was chosen to ensure sufficient magnetic interaction between the nanorod building blocks, such that they could form long chains. Indeed, according to the literature [30], this magnetic field intensity is enough to yield long magnetic rods. Since a nonhomogeneous magnetic field may introduce inhomogeneities (e.g., layered structures at the interface between a ferrofluid and a solid [56]), the magnetic field distribution throughout the sample volume was verified. By obtaining a magnetic field map it was demonstrated that the magnetic field intensity was slightly inhomogeneous across the sample volume (0.45 T near the pole and 0.38 T at the outer side of the vessel). However, this inhomogeneity was not enough to introduce significant changes to the nanoparticle structure [30]. To assess the bulk homogeneity of the synthesized nanoparticles, different sample volumes were used (7 mL, 1.4 mL and 0.35 mL). Changing the sample volume did not alter the synthesized structures, as confirmed by TEM.

The synthesis was carried out for 2–2.5 h. No additional stabilizer was added during the synthesis. Therefore, the interaction between the building blocks and the stabilization of nanoparticles was purely electrostatic and could be tuned by changing the pH. The pH was measured during the synthesis using a SevenMulti pH meter from Mettler Toledo (Columbus, OH, USA). After that, the precipitate was collected by magnetic decantation and washed three times with distilled water. Before TEM and HRTEM experiments, aqueous solutions containing the synthesized nanoparticles were sonicated for 30 min (15 min of pure sonication time, consisting of 5 s pulses followed by 5 s of rest) at a Sonics VCX 500 ultrasonicator (Newtown, CT, USA) in order to break the nanoparticle agglomerates.

### Transmission electron microscopy (TEM)

To prepare the TEM specimens, a drop (10 μL) of a freshly sonicated nanoparticle aqueous solution was deposited onto a 140 mesh Formvar-coated copper grid and and air-dried under ambient conditions. TEM micrographs and electron diffraction patterns were obtained on a LEO 912 AB OMEGA microscope (LEO/Carl Zeiss, Germany) at an accelerating voltage of 100 kV. The reference diffraction pattern of magnetite was obtained from commercially available nanoparticles (ABCR, product number AB304117) with a diameter range of 20–50 nm. Details of the TEM experimental procedures were described elsewhere [57].

### High-resolution transmission electron microscopy (HRTEM)

To prepare the specimens for HRTEM measurements, the samples were manually applied onto the lacey carbon-coated side of the 300 mesh copper grid and air-dried under ambient conditions. The particle morphology was characterized by TEM and scanning TEM (STEM). The specimens were examined under a TITAN 80-300 microscope (FEI, USA) equipped with a Schottky field emission gun, a spherical aberration corrector (Cs probe corrector), a direct detection camera (Falcon II, FEI, USA), and an EDX spectroscopy system (EDAX, USA). The TEM was operated at 300 kV in the STEM bright-field mode. The details regarding the HRTEM equipment and the experimental techniques used were described elsewhere [58–59]. Image processing was performed using the Digital Micrograph (Gatan, USA) and TIA (FEI, USA) software.

### Image processing

The electron micrographs were processed by ImageJ software in order to obtain distances and angles between crystal planes, radial-averaged integrals of electron diffraction patterns and to plot the histograms with the nanoparticle size distribution [60]. Baseline subtraction and peak integration of the radial-averaged diffraction patterns were performed using Origin 8.5. To calculate the area and FWHM, the peaks were fitted by Gaussian functions.

### Raman spectroscopy

The crystalline structure and phase composition of the iron oxide nanoparticles were investigated using a Horiba Jobin Yvon micro-Raman spectrometer (LabRam HR800, Villeneuve-d'Ascq, France) equipped with 100× magnification lens. The copper substrates were dip-coated in the sample solutions and dried under ambient conditions. The measurements of the produced films were conducted at room temperature and atmospheric air. A He–Ne laser (λ = 632.8 nm) was used to excite the Raman scattering. The irradiation power density on the sample was continuously decreased (using neutral density filters and beam defocusing) until no further changes were observed in the obtained spectra. A power of ≈0.2 mW on the sample and a laser spot diameter of ≈30 μm were sufficient to avoid any structural changes or phase degradation in the samples. Under these conditions, the typical total acquisition time to obtain a spectrum with good signal-to-noise ratio was several hours.

## Supporting Information

File 1 EDX spectrum, TEM micrographs, histogram of nanoparticle diameters, and scheme of the experimental setup.
