# Supplementary material for: Magnetic-field-assisted synthesis of anisotropic iron oxide particles: Effect of pH
Source: Beilstein J Nanotechnol. 2020 Aug 17;11:1230–41. doi: 10.3762/bjnano.11.107 (PMC7445396; doi:10.3762/bjnano.11.107)
Supplement: File 1 — EDX spectrum, TEM micrographs, histogram of nanoparticle diameters, and scheme of the experimental setup. [file Beilstein_J_Nanotechnol-11-1230-s001.pdf]

BEILSTEIN JOURNAL OF NANOTECHNOLOGY

Supporting Information

for

Magnetic-field-assisted synthesis of anisotropic iron oxide particles: Effect of pH

Andrey V. Shibaev, Petr V. Shvets, Darya E. Kessel, Roman A. Kamyshinsky, Anton S. Orekhov, Sergey S. Abramchuk, Alexei R. Khokhlov and Olga E. Philippova

Beilstein J. Nanotechnol. **2020,** *11,* 1230–1241. doi:10.3762/bjnano.11.107

EDX spectrum, TEM micrographs, histogram of nanoparticle diameters, and scheme of the experimental setup

License and Terms: This is a supporting information file under the terms of the Creative Commons Attribution License (http://creativecommons.org/licenses/by/4.0). Please note that the reuse, redistribution and reproduction in particular requires that the authors and source are credited.
The license is subject to the *Beilstein Journal of Nanotechnology* terms and conditions: (https://www.beilstein-journals.org/bjnano)

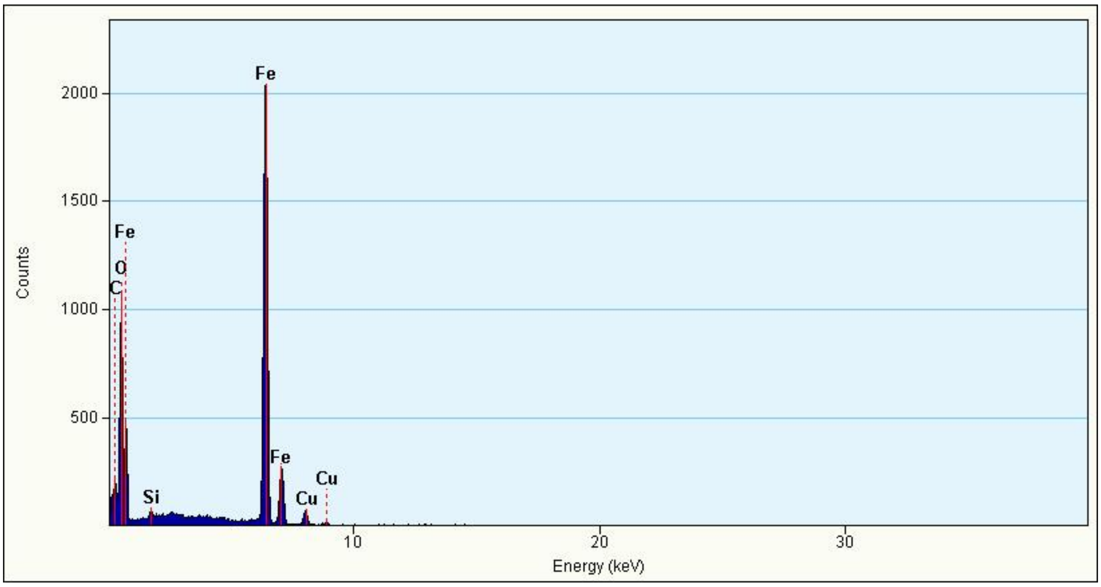

Figure S1: Energy-dispersive X-ray (EDX) spectrum for the sample synthesized at 1.3 M NaOH (*R* = 2.1) under magnetic field of 0.4 T at 20 °C.

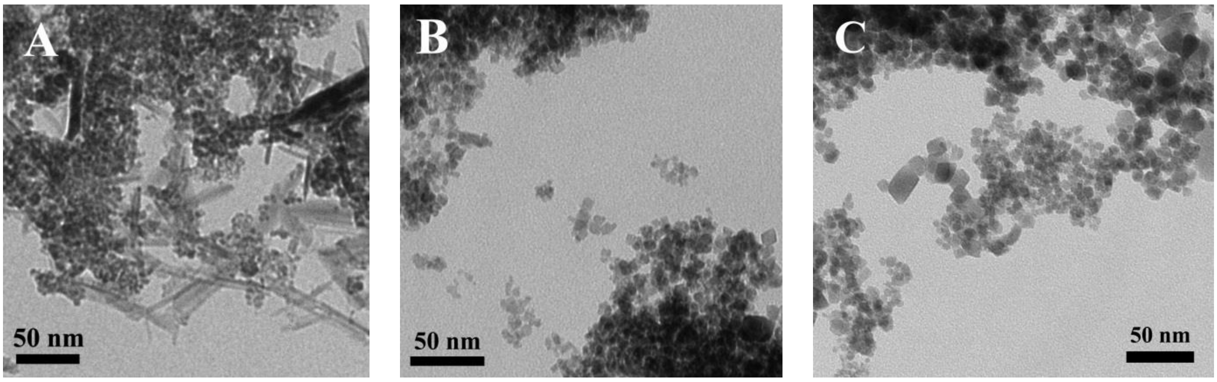

Figure S2: TEM micrographs of nanostructures synthesized at different molar ratios *R* of OH^- ions to the total amount of iron (Fe^{3+} and Fe^{2+}) ions in the mixture: 2.4 (A), 2.8 (B), 3.3 (C) under magnetic field of 0.4 T at 20 °C. pH of the samples after synthesis: 6.3 (A), 9 (B), 12 (C).

Table S1: Content of different atoms calculated from the EDX spectrum.

**Atom**	**Weight %**	**Atomic %**
O	23.7	44.8
Fe	65.8	35.6
C	6.9	17.5
Cu	2.8	1.3
Si	0.7	0.7

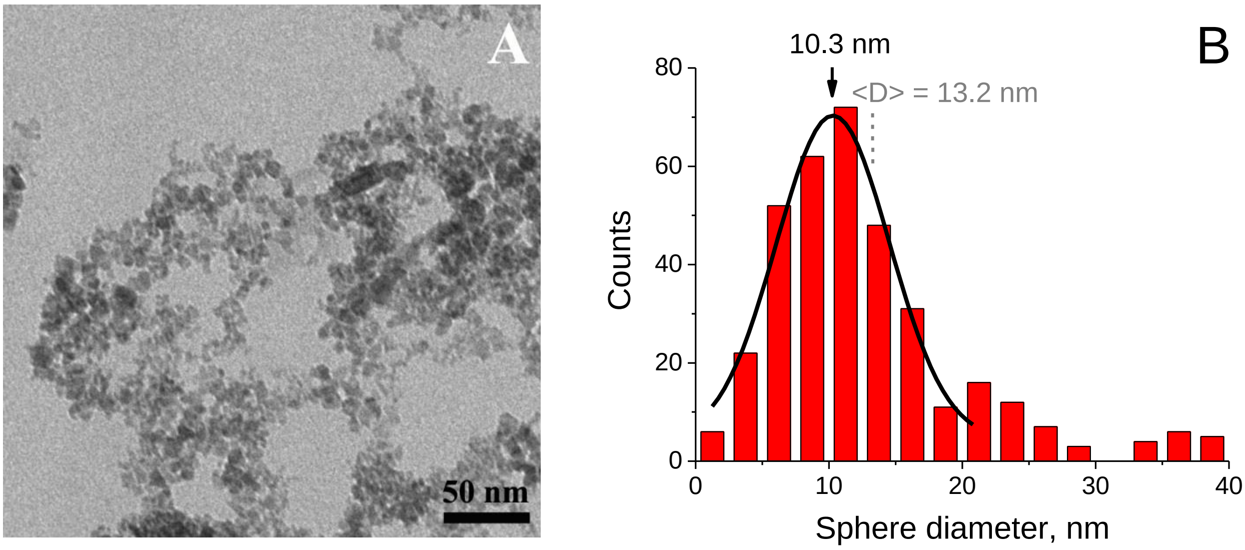

Figure S3: TEM micrograph (A) and histogram of nanoparticles diameters (B) for the sample synthesized at 5 M NaOH (*R* = 8) in the absence of magnetic field at 20 °C.

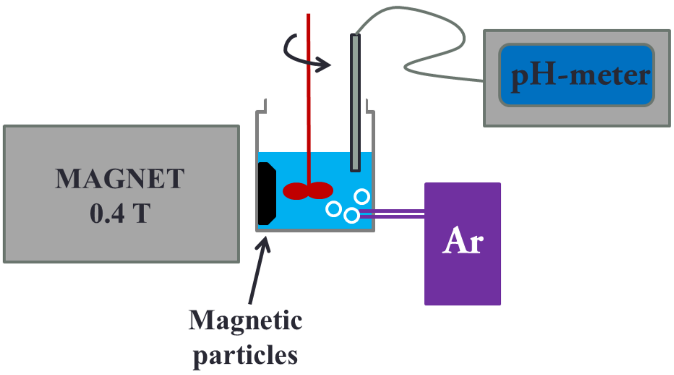

Figure S4: Experimental setup used for the synthesis of magnetite nanorods.
